# Molecular mechanisms and Biological Functions of Autophagy in Endometrial Diseases

**DOI:** 10.7150/ijms.122545

**Published:** 2025-10-27

**Authors:** Jin Xie, Risheng She, Lu Wang, Muhua Yi, Mingjie Dai, Mingxiu Wu, Xianxiu Qiu, Xiaojun Yang

**Affiliations:** 1 Department of Obstetrics and Gynecology, The Tenth Affiliated Hospital, Southern Medical University (Dongguan People's Hospital), Dongguan, 523058, PR China.; 2 Institute of Precision Cancer Medicine and Pathology, School of Medicine, Jinan University, Guangzhou, 510000, PR China.; 3 School of Basic Medicine, Guangdong Medical University, Dongguan, 523808, PR China.

**Keywords:** autophagy, endometriosis, endometritis, endometrial cancer, molecular mechanism

## Abstract

Autophagy is a highly conserved cellular process crucial for maintaining cellular homeostasis by degrading damaged organelles and misfolded proteins. Emerging evidence highlights its pivotal role in endometrial diseases, including endometriosis, endometritis, and endometrial cancer, where dysregulated autophagy contributes to pathogenesis through mechanisms such as altered hormone signaling, inflammation, and metabolic reprogramming. In this review, we comprehensively summarize the molecular machinery of autophagy, its regulatory networks, and its dual roles in endometrial physiology and pathology. Furthermore, we discuss the molecular mechanisms underlying autophagy in endometrial diseases, and the therapeutic potential of targeting autophagy pathways. By integrating recent advances, this review provides insights into autophagy's complex interplay with endometrial diseases and its implications for future research and therapeutic applications.

## Introduction

Endometrial diseases, including endometriosis, endometritis, and endometrial cancer, are prevalent gynecological disorders affecting approximately 10%-15% of women of reproductive age worldwide. Despite advances in diagnosis and treatment, the underlying mechanisms of these diseases remain incompletely understood. Autophagy is a catabolic process that maintains cellular homeostasis by degrading damaged organelles and misfolded proteins. It has emerged as a critical player in endometrial pathophysiology, influencing processes ranging from hormonal regulation to immune responses and tumor progression.

Recent studies have revealed that autophagy exhibits dual roles in endometrial diseases, acting as both a protective mechanism and a contributor to pathology. For instance, in endometrial cancer, it can either suppress tumorigenesis or facilitate immune evasion depending on the molecular context. These divergent effects are mediated through its complex crosstalk with signaling pathways, including AMPK/mTOR, Wnt/β-catenin, and hormone levels, underscoring the complexity of autophagy regulation in endometrial tissues. This review overviews current understanding on the molecular mechanisms of autophagy in endometrial diseases, emphasizing its interplay with other signaling pathways and the therapeutic potential.

## Autophagy

Autophagy is a highly conserved cellular metabolic process. In the process, protein aggregates and damaged cytoplasmic organelles are wrapped by a double membrane structure termed autophagosomes, which then fuse with lysosomes to form autolysosomes, and the contents inside are eventually degraded and released for reuse. Autophagy is an important mechanism for maintaining intracellular homeostasis, and plays an important role in physiological and pathological processes such as defending against pathogen infection, maintaining innate and adaptive immunity, and mediating the pathogenesis and development of diseases 1.

### Core molecular machinery of autophagy

**Figure 1:**
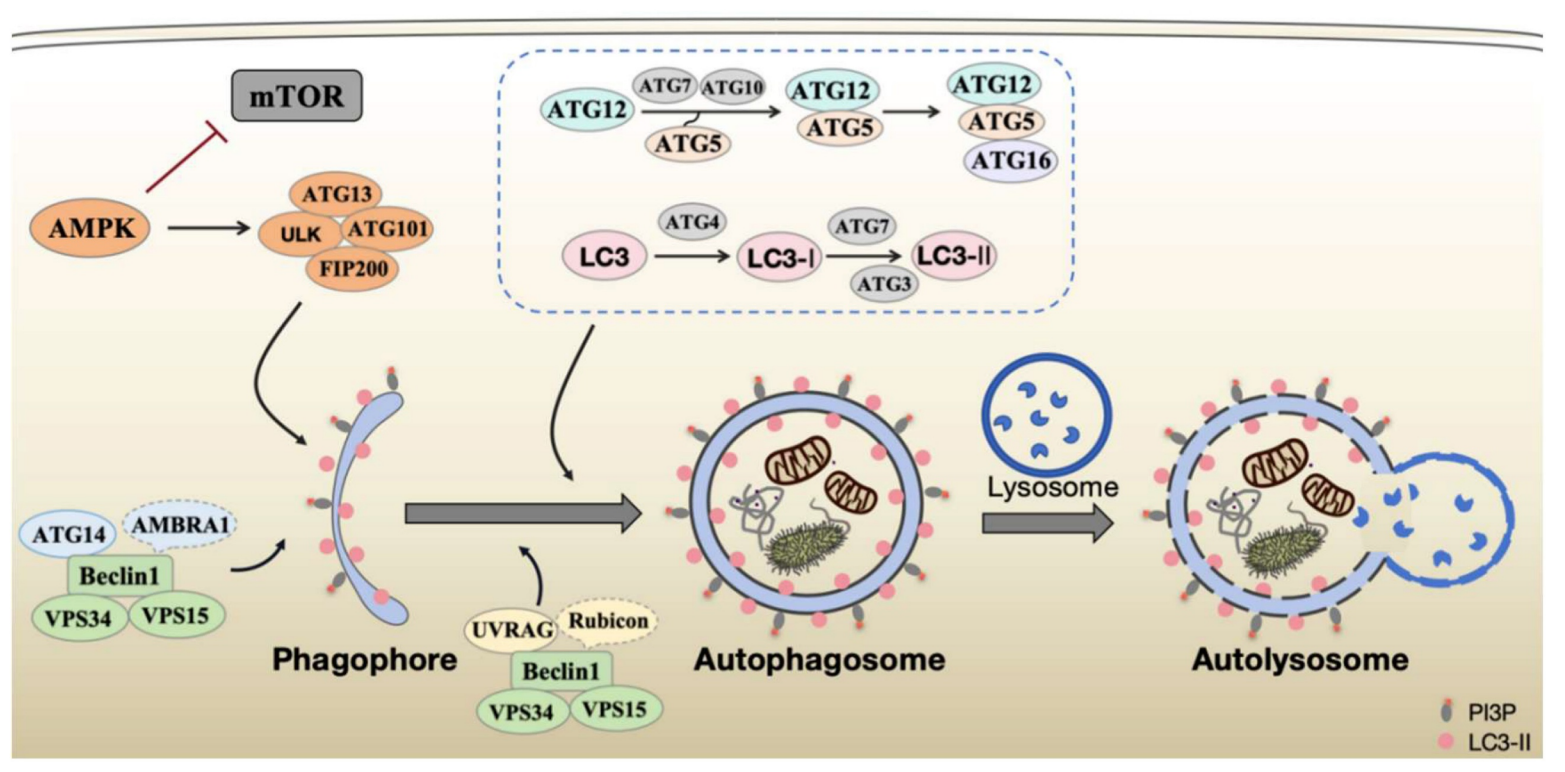
Schematic representation of the autophagy process. The mTOR complex is a central negative regulator of autophagy initiation. AMPK mediated its inhibition and the activation of the ULK1/ATG13/FIP200 complex to initiate phagophore formation. Subsequently, vesicle nucleation is driven by the PI3KC3-C1 (comprising VPS34, VPS15, Beclin1, and ATG14). The expanding phagophore then matures into an autophagosome through the PI3KC3-C2 (containing VPS34, VPS15, Beclin1, UVRAG) and the ATG12 and ATG8/LC3 ubiquitin-like conjugation systems (listed in the box). The autophagosome ultimately fuses with the lysosome to form an autolysosome, where cargos are degraded.

The autophagy process is executed by a sophisticated cascade of protein complexes encoded by autophagy-related genes (ATGs), which work in concert to mediate the sequential stages of autophagy initiation, nucleation, elongation, maturation, and degradation 2 (Figure 1). Recent advances in structural biology and molecular cell biology have significantly enhanced our understanding of the precise architecture and dynamic regulation of these core molecular machines.

#### The ULK1 complex

The molecular regulation of autophagy involves a dynamic interplay between AMP-activated protein kinase (AMPK) and mammalian target of rapamycin (mTOR) signaling pathways. AMPK, a critical cellular energy sensor, stimulates autophagy through direct phosphorylation of ULK1 at Ser317 and Ser777 during glucose deprivation. Conversely, mTOR inhibits autophagic initiation under nutrient-rich conditions by phosphorylating ULK1 at Ser757, thereby disrupting its interaction with AMPK 3. The ULK1 kinase complex, consisting of ULK1, ATG13, FIP200, and ATG101, serves as the molecular switch for autophagy induction by integrating these opposing regulatory signals. Structural studies have revealed that conformational changes in ULK1 serve as the critical determinant of its regulatory function 4. These findings establish a phosphorylation-dependent regulatory mechanism through which cellular energy status controls autophagic flux. Of particular significance, ULK1 has been shown to participate in selective autophagy through phosphorylation of downstream effector molecules, demonstrating its versatile role beyond canonical autophagy initiation 5. Recent cryo-EM studies have further elucidated the molecular details of ULK1 complex assembly and activation, providing new insights into its regulation under various physiological conditions 4.

#### The PI3KC3 complex

The phosphatidylinositol 3-kinase catalytic subunit type 3(PI3KC3), composed of Beclin1 (also known as ATG6), VPS15 and the lipid kinase VPS34, plays an essential role in generating phosphatidylinositol-3-phosphate (PI3P)-enriched membrane platforms for autophagosome nucleation. Emerging evidence indicates that distinct PI3KC3 subcomplexes exhibit stage-specific functions during autophagy progression 6. The PI3KC3 complex I (PI3KC3-C1) containing ATG14 is uniquely involved in autophagy initiation, while the complex II (PI3KC3-C2) containing UVRAG is involved in endosomal sorting and late autophagosome formation 7. Moreover, multiple regulatory proteins including AMBRA1, Rubicon, and Bcl-2 family members can precisely modulate complex activity through dynamic interactions with Beclin1. Notably, post-translational modifications of PI3KC3 components, particularly phosphorylation and ubiquitination of Beclin1, have been identified as crucial mechanisms for fine-tuning autophagy activity in response to diverse cellular stresses 8.

#### The ATG12 and ATG8/LC3 ubiquitin-like conjugation systems

The ATG12-ATG5-ATG16L1 complex, together with ATG8, or its mammalian homolog microtubule-associated protein 1 light chain 3 (LC3), constitutes the molecular foundation for autophagosomal membrane expansion and closure. In the ATG12 system, ATG7 (E1-like enzyme) activates ATG12 through ATP-dependent thioester bond formation, followed by transfer to ATG10 (E2-like enzyme) and subsequent covalent conjugation to ATG5 via isopeptide linkage. The resulting ATG12-ATG5 complexes dimerize with ATG16L to form functional units. For LC3 processing, precursor cleavage by ATG4 exposes a C-terminal glycine, enabling sequential activation by ATG7 and ATG3 (E2-like enzyme) for conjugation of phosphatidylethanolamine (PE). The mature LC3-PE conjugate (LC3-II) becomes membrane-associated, marking autophagic structures 9. Structural studies have recently uncovered the precise assembly mechanism and three-dimensional organization of these conjugation systems, and the dynamic spatial organization and temporal coordination of autophagy machinery components during autophagosome biogenesis 10-12. Moreover, emerging evidence suggests tissue-specific variations in the composition and regulation of these core complexes, potentially explaining organ-specific differences in autophagy activity. Understanding these molecular details provides crucial insights for developing targeted autophagy modulation strategies in various disease contexts.

The ATG8 family proteins has been shown to play critical roles in cargo recognition during selective autophagy, significantly expanding our understanding of autophagy's molecular diversity 13. Cargo receptors specifically bind to lipidated ATG8 family proteins through conserved LC3-interacting region (LIR) motifs. These 15-20 amino acid sequences bind the LIR docking site (LDS) on ATG8 proteins, facilitating selective autophagy substrate recognition 14. Furthermore, novel regulatory mechanisms involving non-canonical functions of ATG12 and LC3 proteins in membrane trafficking and organelle homeostasis have been recently identified, suggesting broader physiological roles beyond their established functions in autophagy 15, 16.

### Regulatory networks of autophagy

Autophagy is precisely regulated through a sophisticated, multi-layered signaling network that integrates diverse cellular inputs. Elucidating these regulatory mechanisms is essential for understanding pathophysiology of human disease and developing precise therapeutic interventions targeting autophagy modulation 17.

#### Nutrient depletion, energy stress and beyond

The mTOR complex 1 (mTORC1) serves as a central nutrient sensor, regulating autophagy through multiple mechanisms 18. Under amino acid-replete conditions, mTORC1 suppresses autophagy initiation by phosphorylating ULK1 at Ser757 and ATG13. It is also revealed that mTORC1 affected the expression of autophagy-related and lysosomal genes via modulating the nucleocytoplasmic shuttling and transcriptional activity of transcription factor EB (TFEB) 19. Notably, while mTORC1 serves as a central amino acid sensor that coordinately regulates cellular growth, metabolic processes, individual amino acids exhibit differential capacities to activate mTORC1, with leucine and arginine eliciting particularly rapid responses through Rag GTPase-dependent mechanisms 20. In contrast, AMPK reciprocally activates autophagy during energy stress through direct phosphorylation of ULK1 and Beclin1, while simultaneously inhibiting mTORC1 activity 3.

Furthermore, autophagy serves as a critical integration point for diverse stress responses, including Endoplasmic Reticulum stress, oxidative stress, hypoxia, and inflammatory signaling pathways 21.

#### Transcriptional regulation

Beyond these nutrient-sensing pathways, transcriptional regulation further modulates autophagic activity 22. TFEB and the forkhead box O (FOXO) family proteins coordinately regulate autophagy gene expression with lysosomal biogenesis, with additional layers of control provided by epigenetic modifications and non-coding RNAs. Whereas the zinc finger transcription factor ZKSCAN3 functions as a transcriptional repressor of autophagy and lysosomal genes during starvation 23, acting antagonistically to TFEB. Besides, canonical transcription factors including E2F transcription factor 1 (E2F1) and Nuclear Factor-κ-gene Binding (NF-κB) also modulate autophagic activity. Both factors transcriptionally activate Beclin1, while they exhibit opposing effects on the expression of Bcl2 interacting protein 3 (BNIP3), a hypoxia-responsive autophagy inducer. However, E2F1 is also revealed to inhibit mitophagy by enhancing the transcriptional expression of Mitofusin 2 24.

This multilayered regulatory architecture enables precise adaptation to metabolic demands and environmental challenges, with important implications for understanding autophagy's role in various pathological conditions.

#### Hormonal and metabolic regulation of autophagy

Emerging research has revealed that hormones and cellular metabolites constitute another critical layer of autophagy regulation. The pancreatic hormones insulin and glucagon function antagonistically to maintain glucose homeostasis through distinct metabolic and autophagic regulatory mechanisms. Insulin inhibits autophagy through mTORC1-mediated ULK1 phosphorylation and FOXO inactivation. Conversely, α-cell-derived glucagon inactivate mTORC1, releasing ULK1 inhibition to stimulate autophagy and catabolic energy production during fasting. Notably, ULK1 regulates mTORC1 via Raptor phosphorylation, establishing an autoregulatory feedback loop 25 26. This sophisticated interplay between pancreatic hormones coordinates both metabolic flux and autophagic activity depending on nutritional status. Similarly, adipokines such as leptin and adipopectin exhibit opposing effects on autophagy in metabolic tissues 27. Thyroid hormones (triiodothyronine and thyroxine) and reproductive hormones (estrogen and progesterone) further contribute to tissue-specific autophagic regulation 28, potentially explaining sex- and metabolic state-dependent variations in disease susceptibility 29.

At the metabolic level, nicotinamide adenine dinucleotide (NAD^+^) homeostasis plays a pivotal role in autophagy regulation through multiple mechanisms. As an essential redox cofactor, NAD^+^ participates in various metabolic reactions while also serving as a critical substrate for NAD^+^-dependent enzymes. Notably, SIRT1-mediated protein deacetylation, dependent on NAD^+^ availability, modulates several autophagic processes. Additionally, CD38-catalyzed NAD^+^ metabolites contribute to autophagy regulation, demonstrating the multifaceted involvement of NAD^+^ metabolism in maintaining autophagic flux 30. Acetyl-CoA, α-ketoglutarate, and sphingolipids (ceramide and sphingosine-1-phosphate) also modulate autophagy through distinct mechanisms, including protein acetylation, HIF-1α stabilization, and membrane signaling 31-33.

These hormonal and metabolic regulators create a dynamic interface between cellular autophagy status and organismal physiology, offering promising targets for therapeutic intervention in metabolic disorders and age-related diseases.

## Autophagy in the Normal Physiology of the Endometrium

The endometrium represents a uniquely dynamic tissue that undergoes precisely coordinated cyclical changes under the regulation of estrogen and progesterone. Autophagy plays a fundamental role in maintaining endometrial physiology through its dynamic regulation of cellular homeostasis. As a highly regenerative tissue that undergoes cyclic remodeling, the endometrium relies on autophagy to balance cell survival and death, facilitate tissue repair, and mediate the inflammatory responses necessary for successful implantation. During the menstrual cycle, fluctuations in estrogen and progesterone levels modulate autophagic activity to support the endometrium's transition through proliferative, secretory, and menstrual phases. Notably, autophagy contributes to the clearance of damaged organelles and proteins while providing metabolic substrates during periods of rapid tissue growth or breakdown.

Accumulating evidence demonstrates that autophagic activity is indispensable for both endometrial homeostasis and reproductive function. At the molecular level, autophagy coordinates with key reproductive hormones to regulate endometrial function. Through its interplay with mTOR and AMPK pathways, autophagy integrates nutrient sensing with hormonal regulation to optimize endometrial receptivity. Studies have demonstrated that impaired autophagy disrupts uterine gland morphogenesis and stromal cell decidualization, two critical processes for establishing pregnancy 34, 35 . For instance, Genetic ablation of Beclin1 in uterine tissue severely impairs endometrial development and pregnancy establishment. Beclin1-mediated autophagy, rather than apoptosis, is critical for maintaining LGR5^+^/ALDH1A1^+^ endometrial progenitor stem cell populations through regulation of Wnt signaling pathway 34. Furthermore, proper autophagic flux maintains mitochondrial quality control in endometrial cells, ensuring adequate energy production for the metabolically demanding processes of embryo implantation 36.

Optimal autophagic activity supports fertility by creating a receptive endometrial environment through careful regulation of inflammation, vascular remodeling, and cellular turnover. During early pregnancy, autophagy mediates the balance between promoting trophoblast invasion while preventing excessive tissue breakdown. Emerging evidence suggests that autophagy dysregulation may contribute to various reproductive disorders, including implantation failure, recurrent pregnancy loss, and endometriosis 37. Enhanced autophagic activity was observed in human endometrial stromal cells during *in vitro* decidualization. Genetic silencing of key autophagy regulators like ATG5 and ATG7 significantly compromises this differentiation process, confirming their essential role in proper decidualization 38.

While basal autophagy maintains normal endometrial function, emerging research reveals that dysregulation of autophagic flux contributes to various endometrial pathologies. Interestingly, both excessive and insufficient autophagy have been documented in different disease contexts, highlighting the dual role of autophagy in endometrial physiology. This paradoxical involvement suggests that precise autophagic balance is crucial for endometrial health, with deviations in either direction potentially leading to pathological consequences. Current research suggests that pharmacological modulation of autophagy could enhance endometrial receptivity in clinical applications, though the narrow therapeutic window requires precise modulation. Further investigation is needed to fully elucidate how autophagy interacts with hormonal and other signaling to coordinate endometrial function throughout the menstrual cycle and during pregnancy.

## Autophagy in Endometrial Diseases

### Endometriosis

Endometriosis (EMs) is a common proinflammatory gynecological disease characterized by abnormal growth of endometrial tissue outside the uterine cavity. Autophagy has been shown to influence the survival and apoptosis of ectopic endometrial cells, affecting disease progression. Dysregulation of autophagy pathways may enable these misplaced cells to evade normal cell death mechanisms, promoting lesion persistence. Understanding autophagy's role could lead to new therapeutic strategies for managing endometriosis.

EMs affects approximately 10% of reproductive-age women and is associated with infertility 39. The currently widely accepted etiology of endometriosis is the retrograde menstruation theory proposed by Sampson, who hypothesizes that the viable cells and menstrual fragments with retrograde menstruation could migrate, grow, infiltrate into the peritoneal cavity, and cause chronic inflammation. The ectopic endometrium could invade any part of the body. It is most frequently found in ovaries, resulting in the formation of chocolate cysts. While it can also be found in the uterine tubes, uterosacral ligaments, the gastrointestinal tract. The growth of endometrium-like tissue outside of the uterus is responsible for symptoms including chronic pelvic pain, dysmenorrhea, and subfertility. The pathogenesis is complex and involves multiple factors, including genetic predisposition, hormonal imbalance, and immunological disorders 40. At the molecular level, ectopic endometrial tissue in EMs patients exhibits unique biological characteristics. Firstly, enhanced proliferation and anti-apoptosis ability, which is associated with Bcl-2 overexpression and decreased caspase-3 activity 41. Secondly, abnormal inflammatory microenvironment, with increased levels of proinflammatory factors such as IL-6, IL-8, and TNF-α 39. And thirdly, imbalanced estrogen metabolism, with increased local estrogen synthesis and decreased metabolism 42. These pathological and molecular alternations jointly promote the formation and development of ectopic endometrial lesions.

There is no cure for EMs thus far, and treatment mainly focuses on symptom management that could be broadly categorized into two main types: surgical resection and pharmacological therapy. Although inevitably entails some risks related to the complications, surgical resection remains a potential option of treatment given its ability to enhance fertility capability and achieving long-term relief 43. The first-line pharmacological therapy for endometriosis treatment includes non-steroidal anti-inflammatory drugs, progestins, or combined hormonal contraceptives.

Recent studies have highlighted the role of autophagy in the development and progression of endometriosis. It is shown that autophagy contributes to the survival and invasiveness of ectopic endometrial cells. Choi *et al.* reported that autophagy activity is significantly elevated in ectopic endometrial cells compared to normal endometrial cells, and this activated autophagy is associated with enhanced cell survival by removing damaged organelles and proteins 44. The molecular mechanisms underlying autophagy in endometriosis involve the activation of the AMPK/mTOR pathway and the upregulation of autophagy-related genes such as LC3B and Beclin1.

#### Wnt signaling pathways

Wnt signaling pathways could be broadly classified into canonical Wnt signaling (β-catenin dependent) and non-canonical Wnt signaling (β-catenin independent). The Wnt/β-catenin signaling pathway plays a key role in the occurrence and development of endometriosis. It is initiated by the trimeric complex formed by Wnt ligand and its receptor Frizzled (FZD) and LRP5/LRP6 on the cell surface. Frizzled receptor activation mediates the recruitment of the effector protein Dishevelled (Dvl), which in turn recruits GSK3β and other core destruction complex components to the plasma membrane. The destruction complex is inhibited, preventing β-catenin phosphorylation and degradation, resulting in its accumulation and translocation into the nucleus. Then the β-catenin interacts with the T-cell factor/lymphoid enhancer-binding factor (TCF/LEF) family, and drives the transcription of downstream target genes involved in cell proliferation, differentiation, and survival 45.

A recent study constructed a high-resolution Human Endometrial Cell Atlas (HECA) by integrating published and new endometrial single-cell transcriptomics datasets of 63 women with and without endometriosis 46. The results showed that the differentially expressed genes in endometrial cells of endometriosis patients and normal subjects were mainly enriched in the Wnt signaling pathway. The canonical Wnt/β-catenin pathway regulates cell proliferation and differentiation. In endometriosis, abnormal activation of Wnt/β-catenin signaling promotes the proliferation and invasion of ectopic endometrial cells and inhibits apoptosis; while the inhibition of this pathway can reduce cell proliferation and invasion and promote apoptosis.

The non-canonical Wnt signaling includes the planar cell polarity (PCP) and Wnt-calcium (Wnt/Ca^2+^) pathways that function independently of β-catenin and TCF/LEF effectors. Studies have also reported that the aberrant activation of the non-canonical Wnt signaling, notably through upregulation of the Wnt5A ligand in ectopic endometrium, promoted the growth of endometriotic lesions. The Wnt5A-expressing ectopic endometrial stromal cells may send "growth instructions" to surrounding ovarian stromal cells, promoting abnormal cell proliferation, migration and inflammatory response that contribute to the establishment of ectopic lesions 47.

To prevent the aberrant activation of Wnt signaling, regulatory feedback mechanisms between Wnt signaling and different cellular homeostasis pathways including autophagy have been reported at different levels. For instance, β-catenin and Dvl are substrates of selective autophagy for degradation under stress conditions like nutrient deprivation. Upon starvation, β-catenin directly interacts with LC3 through its LC3-interacting region (LIR), and is trafficked into autolysosome for degradation, leading to attenuated β-catenin/TCF-driven transcription and cell proliferation 48. The Dvl protein is a critical Wnt signaling regulator which participates in all three types of Wnt/β-catenin, PCP and Wnt/Ca^2+^ signaling pathways. It is reported that autophagy negatively regulates Wnt signaling by mediating the selective autophagic degradation of Dvl, either through the adaptor protein p62 or direct interaction with LC3. Dvl is ubiquitinated by the Von Hippel-Lindau protein (pVHL), an E3 ligase, and recognized by p62 via its ubiquitin-associated domain. The ubiquitylated Dvl-p62 complex then binds with LC3 through the LIR in p62, thus directing Dvl into the autolysosome for degradation 49. Dvl is also shown to directly interact with LC3 via its LIR motif, and to be degraded by autophagy-lysosome pathway.

#### Hormone levels

Endometriosis is reported to be an estrogen-dependent inflammatory disease that with estrogen and progesterone imbalance in ectopic lesions. it is shown that increased estrogen and decreased progesterone levels synergistically promote the survival of ectopic endometria. Autophagy is involved in regulating hormone balance within the endometrium, particularly estrogen levels. During the normal human secretory phase, estrogen exhibits the inhibitory effect on autophagy while progesterone is a potent autophagy activator for endometrial stromal cells (ESCs) 44. During menstruation cycle, autophagy levels remain low in the proliferative phase but significantly increase at the late secretory phase. This interplay between estrogen and autophagy is disrupted in endometriosis.

Estrogen exerts its effects primarily via estrogen receptor alpha (ERα). ERα is generally overexpressed in endometriosis, and is essential for estrogen-induced cell survival, proliferation, and inflammation in endometriotic lesions. Autophagy plays a critical role in maintaining estrogen signaling in endometriosis by mediating ERα degradation. Although ERα is primarily degraded through the ubiquitin-proteasome system, its levels also reduce by the autophagy-lysosome pathway 50, 51.

#### Apoptosis and others

It has been described that the autophagy level was decreased in ectopic endometria, and that the reduced autophagy activity promoted the survival of ectopic endometria, which was related to apoptosis inhibition. Notably, autophagy activators would inhibit the growth of endometriotic lesions, reduce the lesions' volume, area and diameter 52. Choi* et al.* revealed that progestin (dienogest) treatment increased LC3-II and cleaved caspase-3 expression, and stimulated autophagosome formation in estrogen-treated endometriotic cyst stromal cells (ECSCs). These autophagy-inducing effects were accompanied by inhibition of AKT, extracellular signal-regulated kinase (ERK), mammalian target of rapamycin (mTOR) signaling. While treatment with dienogest and 3-methyladenine (an autophagy inhibitor) in ECSCs showed impaired autophagy and apoptosis activity as reflected by the decreased levels of LC3-II and cleaved caspase-3. These findings indicated that dienogest treatment in estrogen-treated ECSCs could stimulate autophagy by suppressing AKT, ERK1/2, and mTOR activity, further promote apoptosis in endometriosis 44.

In addition to apoptosis, autophagy is involved in the hormonal homeostasis of endometriosis through its effects on inflammation and oxidative stress. Mei *et al.* reported that reduced autophagy activities were observed in ectopic and eutopic endometrial stromal cells from women with endometriosis, and that the autophagy level was strongly negatively correlated with the concentration of CXCL12 in ESCs. Estrogen-mediated suppression of autophagy is dependent on the interaction between CXCL12 and its receptor CXCR4, which could be abrogated by the anti-CXCR4 neutralizing antibody and by the NF-κB inhibitor 53.

### Adenomyosis

Similar to endometriosis, adenomyosis is also an estrogen-dependent gynecological disorder but characterized by the invasion of endometrial tissue into the myometrium, forming ectopic endometrial lesions and leading to uterine enlargement, pain, abnormal bleeding, and infertility. Recent research suggests that autophagy may facilitate the invasive behavior of endometrial cells in adenomyosis by modulating cellular survival and tissue remodeling 54. Dysregulation of autophagy could contribute to the pathological changes in the disease.

Increased IL-18R expression in eutopic endometrium from adenomyosis patients suggests chronic inflammation contributes to lesion development 55. Experimental evidence demonstrates that lipopolysaccharide (LPS)-induced toll-like receptor (TLR) signaling activation promotes stromal cell proliferation and invasion via growth factor-mediated inflammation 56. Moreover, nonsteroidal anti-inflammatory drugs (NSAIDs) provide partial symptomatic relief in adenomyosis, further implicate inflammatory pathways, such as IL-18 and TLR4, in adenomyosis pathogenesis 57. Emerging evidence suggests that the eutopic endometrium in adenomyosis exhibits enhanced cell proliferation and migration, elevated epithelial-to-mesenchymal transition (EMT), increased apoptotic resistance, and altered extracellular matrix (ECM) function. Autophagy plays overlapping but distinct roles in the pathogenesis of endometriosis and adenomyosis. In addition to regulating estrogen signaling and cell survival, autophagy plays a crucial role in adenomyosis pathogenesis by modulating cell migration and fibrosis.

#### Epithelial-to-mesenchymal transition

EMT refers to the biological process in which epithelial cells lose their cell-cell adhesion and polarity, transforming into mesenchymal cells with increased mesenchymal marker expression like N-cadherin. It is reported that the level of E-cadherin was downregulated in epithelial cells in human adenomyosis, in comparison with control endometrium 54. EMT helps cell migration and invasion, which is implicated in the development and progression of adenomyosis. Mechanistically, EMT facilitates endometrial cell detachment from their native site and subsequent myometrial invasion, leading to ectopic lesion formation. It also promotes fibrotic tissue accumulation in the myometrium, a hallmark of adenomyosis.

Various signaling pathways regulate EMT in adenomyosis, including transforming growth factor -β (TGF-β), focal adhesion kinase (FAK), and Wnt/β-catenin cascades. Notably, adenomyotic endometrium shows elevated nuclear β-catenin levels compared to normal tissue, accompanied by cadherin switching from E-cadherin to N-cadherin. In the mouse model of adenomyosis, it is shown that β-catenin activation induces EMT by upregulating levels of transcription factors including Snail and ZEB1, while suppressing E-cadherin expression 54. In addition to mediating the autophagic degradation of β-catenin, autophagy could also regulate EMT process by degrading E-cadherin, via p62/SQSTM1-mediated selective autophagy 58. Defective autophagy is correlated with enhanced EMT and endometrial fibrosis in intrauterine adhesion (IUA) patients. This autophagic impairment is associated with the reduced iodothyronine deiodinase 2 (DIO2) expression. *In vitro* studies revealed that pharmacological modulation of autophagy significantly influenced EMT progression in endometrial epithelial cells. Specifically, chloroquine (CQ)-mediated inhibition of autophagy enhanced this process, whereas rapamycin-induced activation attenuated it. Mechanistic investigations demonstrated that DIO2 knockdown impaired autophagic flux and promoted EMT through MAPK/ERK-mTOR pathway activation. Conversely, DIO2 overexpression or triiodothyronine (T3) administration restored autophagic activity and partially reversed EMT. Consistently, in a murine model of IUA, enhancing autophagy through rapamycin or T3 treatment alleviated fibrosis, while its inhibition by CQ promoted fibrotic progression 59.

#### Fibrosis

Fibrosis represents a key pathological feature of adenomyosis, which is developed through repeated cycles of tissue injury and repair (TIAR). The TGF-β signaling pathway serves as a major driver of this process by inducing EMT, collagen deposition, and smooth muscle metaplasia (SMM) 60. At the molecular level, TGF-β activates Smad proteins via TGF-β receptors (TGFBR), triggering downstream gene expression that promotes fibrotic lesion formation.

Elevated TGF-β1 levels have been consistently observed in adenomyosis patients, with experimental evidence demonstrating that TGF-β1 inhibition reduces uterine collagen accumulation and attenuates disease-associated fibrosis 61. In contrast, another study reported that no significant elevation in the mean TGF-β1 expression in adenomyosis patients, suggesting potential variability in TGF-β1 involvement across different patient populations. Whereas experimental evidence reveals that elevated TGF-β2 levels are observed in both ectopic and eutopic endometrium of adenomyosis patients compared to controls. The TGF-β2 expression is directly regulated by β-catenin, with a significant positive correlation exists between β-catenin and TGF-β2 protein levels, suggesting TGF-β2 serves as a critical mediator in adenomyosis pathogenesis through β-catenin activation 62.

TGF-β enhances the autophagy flux and elevates the expression levels of autophagy-related genes. Zehender *et al.* reported that TGF-β enhances its profibrotic effects through epigenetically regulated autophagy activation. Mechanistically, TGF-β downregulates the H4K16 histone acetyltransferase MYST1 via Smad3, and subsequently upregulating key autophagy components like ATG7 and Beclin1. This autophagy induction in fibroblasts induces collagen secretion, and is necessary and sufficient to trigger tissue fibrosis. Restoring MYST1 expression counteracts TGF-β-mediated autophagy stimulation. Importantly, inhibiting aberrant autophagy blocks TGF-β-driven fibroblast activation and alleviates fibrosis in experimental models 63. Autophagy modulates TGF-β signaling by stabilizing membrane raft-localized TGF-βRII, thereby limiting receptor internalization and subsequent R-Smad activation. While autophagy inhibition attenuated TGF-β-dependent cadherin switching, stress fiber assembly, and migratory capacity 64. Genetic evidence demonstrates that LC3B knockout mice exhibited elevated renal collagen accumulation and mature TGF-β levels following ureteral obstruction. Similarly, Beclin1 haploinsufficient animals showed enhanced fibrotic deposition. Both LC3 deficiency and autolysosomal blockade using bafilomycin A1 led to increased mature TGF-β accumulation 65. While how autophagy regulates TGF-β driven fibrosis in adenomyosis remains to be further investigated.

### Endometritis

Endometritis, an inflammatory disorder of the endometrium, primarily results from bacterial infections and manifests as chronic pelvic pain, abnormal uterine bleeding, and infertility. It can be classified as acute or chronic based on disease progression. Acute endometritis typically involves pathogens such as *Escherichia coli*, *Streptococcus*, and *Staphylococcus*, while chronic endometritis (CE) stems from persistent low-grade infections. CE alters the endometrial immune milieu, significantly impairing receptivity and adversely affecting reproductive outcomes, including implantation failure and recurrent pregnancy loss. Disease progression depends largely on the endometrium's ability to mount an appropriate immune response against microbial invasion 66. Autophagy plays a critical role in the immune response by regulating the degradation of pathogens and modulating inflammatory processes.

Endometritis is characteristized by several pathological changes, including (1) destruction of endometrial tissue structure, and separation of epithelial cells and stroma; (2) infiltration of plasma cells in the stroma; (3) increased vascular permeability and endometrial surface mucosal edema 67. Plasma cell infiltration serves as a key diagnostic marker for CE, with B cell populations demonstrating characteristic alterations in this condition. While endometrial B cells typically constitute less than 1% of leukocytes and primarily localized as isolated cells or small aggregates in the basal layer, CE induces significant expansion through microbial pathogen-triggered mechanisms^68^. During CE pathogenesis, microbial components (e.g., LPS) stimulate endometrial epithelial cells to overexpress chemotactic mediators including CXCL1, CXCL13 and E-selectin^69^. This chemokine upregulation promotes extensive B cell recruitment from peripheral circulation, resulting in lymphocyte infiltration throughout both functional and basal endometrial layers, with occasional glandular epithelium penetration.

#### NF-κB and MAPK signaling

Multiple signaling pathways, including NF-κB, mitogen-activated protein kinase (MAPK), and PI3K/Akt/mTOR, critically regulate endometrial inflammatory responses and cellular processes. Their dysregulation contributes to endometritis pathogenesis. At the molecular level, pattern recognition receptors (PRRs), such as TLRs and Nod-like receptors (NLRs), play a crucial role in the inflammatory response initiation by recognizing pathogens or cellular damage. They activate NF-κB and MAPK signaling cascades, and subsequently induce excessive production of pro-inflammatory cytokines including IL-1β, IL-6 and TNF-α^70^, ^71^.

The NF-κB pathway serves as a master inflammatory regulator, which could be activated by bacterial components, cytokines, or hormonal signals in endometritis. This activation triggers excessive production of proinflammatory cytokines and chemokines, exacerbating tissue damage when dysregulated. Similarly, MAPK cascades, including extracellular signal-regulated kinase (ERK), c-Jun N-terminal kinase (JNK), and p38, respond to inflammatory stimuli through PRRs, amplifying cytokine release and promoting the progression of endometritis. Normally, the NF-κB and MAPK signaling cascades coordinated to amplify the inflammatory response by inducing the production of pro-inflammatory cytokines 72.

Growing evidence suggests that impaired autophagy contributes to endometritis pathogenesis. It is demonstrated that the autophagy molecular marker LC3-II is upregulated while mTORC1 activity is suppressed in patients with CE. Concurrently, IL-17 levels are significantly elevated but IL-10 and TGF-β expression are reduced. These findings suggest CE promotes a Th17-dominant over Treg immune response in the endometrium, likely mediated through mTORC1-dependent autophagy dysregulation 73.

In bovine endometrial epithelial cells (BEECs), autophagy is suppressed upon LPS treatment, with NF-κB pathway activation and elevated IL-6, IL-8, and TNF-α mRNA levels. Pharmacological inhibition of autophagy using CQ exacerbated NF-κB signaling and proinflammatory cytokine expression in LPS-treated BEECs, whereas rapamycin-induced autophagy activation exerted opposite effects 74. These findings suggest autophagy activation may represent a potential therapeutic strategy for endometritis management. Actually, it is reported that melatonin treatment exhibites anti-inflammatory effects in LPS-induced endometritis by enhancing autophagy and suppressing NLRP3 inflammasome activation 75. Similarly, astragaloside IV (AS IV) pretreatment attenuates LPS-stimulated IL-1β and TNF-α production while upregulating autophagy markers (LC3-II/LC3-I and Beclin1). This protective effect can be reversed by 3-Methyladenine (3-MA), an autophagy inhibitor, confirming AS IV protected against endometrial inflammation by autophagy induction. Further mechanistical studies indicated that AS IV activates the autophagy via cAMP-PRKA-AMPK-SIRT1 cascade 76.

#### Pathogen infection

Autophagy plays a dual role in endometritis pathogenesis, serving as both a protective and pathogenic mechanism. Representing as an essential antimicrobial defense mechanism in uterine infections, it mediates the engulfment and degradation of pathogens, such as *Escherichia coli,* within endometrial epithelium through a process termed “xenophagy”. This process not only restricts cytosolic bacterial proliferation but also attenuates inflammatory responses and tissue damage by eliminating pathogenic components. Conversely, autophagic dysregulation exacerbates disease progression through establishment of the immunosuppressive microenvironment. Endometrial cells from patients often exhibit dysregulation of both autophagic and innate immune functions.

It is reported that* Escherichia coli* infection in bovine endometrial epithelial cells induces cytoplasmic calcium overload and mitochondrial impairment. Elevated cytosolic Ca²⁺ disrupted mitochondrial function and activated endoplasmic reticulum stress (ERS), exacerbating bovine endometritis pathogenesis. Notably, autophagy activation suppressed inflammatory cytokine release (IL-1β, IL-18), mitochondrial damage markers (cytochrome c, ATP), ERS-related proteins, and calcium dysregulation. These results establish autophagy as a key modulator of *Escherichia coli*-induced bovine endometritis via coordinated regulation of calcium homeostasis, mitochondrial function, and ERS 77.

It is also revealed that *Trueperella pyogenes* virulence factor pyolysin (PLO) suppresses LPS-induced innate immunity in goat endometrial stromal cells during endometritis. Pharmacological and genetic analyses reveal that recombinant PLO activates autophagy to downregulate inflammatory gene expression via NLRP3 inflammasome inhibition. Additionally, ATF6 is identified as the dominant regulator of PLO-mediated inflammatory responses. These findings suggest PLO's capacity to establish an immunosuppressive microenvironment for other pathogens invasion through coordinating autophagy and ATF6 signaling pathways 78.

A recent study investigated the role of host cell autophagy in *Neospora caninum* infection using the caprine endometrial epithelial cell model 79. It is found that infection significantly upregulates LC3-II expression and autophagosome formation, while decreasing the autophagic substrate p62 levels. Further experiment using adenoviral mCherry-GFP-LC3B transfection confirms enhanced autophagic flux in endometrial epithelial cells. Pharmacological modulation reveales that mTOR inhibition by rapamycin promotes tachyzoite proliferation, whereas autophagy suppression by CQ hindered parasite replication. Mechanistically, *Neospora caninum* triggers autophagy by suppressing mTOR phosphorylation 79.

### Endometrial cancer

Endometrial cancer (EC) has emerged as one of the most prevalent gynecological malignancies in developed countries, with increasing incidence in recent decades probably correlate with extended life span and rising prevalence of obesity 80. Numerous studies have established that autophagy is intricately involved in multiple aspects of tumorigenesis and metastasis 81 82. The relationship between autophagy and cancer is complex and context-dependent, acting as both a tumor suppressor and a tumor promoter 82. On one hand, autophagy maintains genomic stability by eliminating damaged organelles and misfolded proteins, thereby exerting tumor-suppressive effects. On the other hand, under the metabolic stress of the tumor microenvironment, autophagy provides crucial metabolic support for cancer cell survival and contributes to chemoresistance 83. This double-edged sword effect highlights the complex and dynamic nature of autophagy in cancer progression, emphasizing the significance of elucidating the precise regulatory mechanisms governing autophagic activity.

Based on distinct clinicopathological characteristics, EC is classified into two major subtypes: estrogen-dependent type I (endometrioid adenocarcinoma, accounting for approximately 80% of cases) and estrogen-independent type II (non-endometrioid histologies including serous and clear cell carcinomas). Type I EC typically exhibits more favorable prognosis, whereas type II tumors demonstrate aggressive biological behavior and poorer clinical outcomes 84.

The molecular pathogenesis of EC involves complex genetic and epigenetic alterations that drive malignant transformation through multiple interconnected pathways. First, somatic mutations in PTEN, PIK3CA, and KRAS lead to constitutive activation of the PI3K/AKT/mTOR pathway, which promotes uncontrolled cellular proliferation, metabolic reprogramming, and resistance to apoptosis through downstream effectors 85. Secondly, microsatellite instability (MSI) and mismatch repair (MMR) defects. Thirdly, type II EC exhibits specific molecular alterations in TP53 mutations and HER2/neu amplification, which associated with enhanced metastatic potential and therapeutic resistance 86. Recent advances in multi-omics research have further enhanced our comprehension of EC molecular heterogeneity, while simultaneously identifying novel disease-specific biomarkers. These comprehensive analyses yield critical prognostic indicators and reveal potential therapeutic targets, thereby facilitating the development of precision medicine for EC treatment 87.

#### The crosstalk of autophagy and immune responses

The crosstalk between autophagy and the immune system plays a critical yet complex role in the tumor microenvironment of EC, significantly influencing anti-tumor immunity. Autophagy acts as a key regulator of innate immune responses and antigen presentation. It maintains immune homeostasis by clearing damage-associated molecular patterns (DAMPs) and pathogen-associated molecular patterns (PAMPs). A prime example is mitophagy, which removes damaged mitochondria to prevent the accumulation of mitochondrial DAMPs, thereby suppressing type I interferon production and inflammasome activation, and subsequently reducing the secretion of pro-inflammatory cytokines like IL-1β and IL-18 88. Consequently, autophagy deficiency can lead to a hyperinflammatory tumor microenvironment. This inflammatory response may initially inhibit tumor growth. However, by failing to clear the damaging stimuli, it ultimately promotes tumorigenesis by sustaining a chronic state of inflammation.

Furthermore, autophagy is indispensable for the development, survival, and function of lymphocytes. In T cells, autophagy is crucial for the survival of naive cells and is upregulated upon T-cell receptor (TCR) stimulation to support their activation and proliferation. This process is mediated by AMPK-ULK1 signaling, and genetic deletion of key autophagy genes like ATG5 or ATG7 leads to a failure in T-cell expansion 101. Similarly, autophagy is involved in the development and differentiation of B cells, with studies showing that ATG5 is required for the development of specific B-cell subsets and for effective antibody production upon activation 89, 90.

#### Autophagy in tumor suppression

It is revealed that Beclin-1-dependent autophagy restricted malignant progression by promoting tumor cell death. In Becn1F121A/F121A knock-in mice with mutations disrupting the Beclin1-Bcl-2 interaction, display elevated basal autophagic activity and consequently develop fewer age-related tumors compared to wild-type counterparts 91. However, genomic analyses by Lebovitz *et al.* identified recurrent somatic mutations in core autophagy genes (ATG4C, RB1CC1/FIP200, ULK4) within endometrial carcinoma specimens. These genetic alterations, particularly prevalent in type I endometrial cancers, may compromise autophagic activity and its tumor-suppressive functions 92.

Recent mechanistic studies have identified specific pathways through which autophagy influences endometrial cancer progression. For instance, the ubiquitin-conjugating enzyme UBE2C promotes endometrial cancer progression by suppressing autophagy through epigenetic regulation. Cellular studies revealed that UBE2C knockdown induces autophagy. Mechanistically, UBE2C catalyzes K48-linked ubiquitination of SIRT1, triggering its proteasomal degradation. This process reduces H4K16 deacetylation, thereby epigenetically silencing autophagy-related genes. The xenograft models show that UBE2C overexpression promotes tumor growth, while the autophagy activator rapamycin treatment reverses UBE2C-driven tumor growth and apoptosis resistance both *in vitro* and* in vivo* 93. Moreover, elevated PFKFB3 expression in EC cell lines is demonstrated to be correlated with chemoresistance. Knockdown of PFKFB3 or treatment with its inhibitor by PFK158 significantly suppresses proliferation while sensitizing EC cells to carboplatin and cisplatin. Mechanistic studies reveal that combined treatment of PFK158 and carboplatin/cisplatin in chemoresistant EC cell lines induces apoptotic and autophagic cell death through Akt/mTOR pathway suppression 94. These findings suggest activation of autophagy could inhibit EC progression and enhance its chemosensitivity.

#### Autophagy in tumor promotion

Conversely, autophagy can also facilitate immune evasion in endometrial cancer. Specifically, NLRC5 is an innate immune regulator and major MHC-I transactivator that plays a critical role in tumor antigen presentation. Its downregulation in EC facilitates immune evasion and correlates with immunotherapy resistance. Zhan *et al.* demonstrated that autophagy suppressed both NLRC5 expression and subsequent MHC-I gene activation. Notably, a novel interaction between LC3 and NLRC5 is identified to inhibit MHC-I antigen presentation *in vitro* and* in vivo*. These findings reveal an autophagy-mediated immune evasion mechanism in EC through LC3-dependent NLRC5 suppression 95. Furthermore, it is demonstrated that SIRT1 plays a pivotal oncogenic role in endometrial cancer through its regulation of distinct autophagy pathways. Elevated SIRT1 expression in EC tissues and cell lines promotes tumor progression via two interconnected mechanisms: by directly deacetylating LC3 to enhance autophagic activity, and through physical interaction with FOXO3 to activate the BNIP3-PINK1/Parkin mitophagy axis. The coordinated action of these pathways drives key malignant phenotypes including increased proliferation, invasion, and hormone resistance, as evidenced by both *in vitro* and* in vivo* models 96, 97.

Clinical correlations also support these experimental findings. In EC tumor tissues, elevated protein levels of cyclin D1, Beclin1, ATG5, ATG7, and LC3 are observed, and upregulation of cyclin D1 is significantly associated with lymph node metastasis in EC. Positive correlations are identified between cyclin D1 and autophagy markers Beclin1 and ATG5, suggesting cyclin D1-mediated autophagy activation may facilitate lymph node metastasis in endometrial cancer 98. Importantly, therapeutic interventions combined with autophagy suppression show promising synergistic anti-tumor effects. A recent study investigated the therapeutic potential of ICG-001, a β-catenin inhibitor, in endometrial cancer using patient-derived xenograft organoids and primary cell models. Experimental results demonstrated that ICG-001 effectively suppressed tumor growth and while simultaneously inducing autophagic activity. Mechanistic studies reveal that combined treatment with ICG-001 and ATG5 silencing synergistically inhibits cancer cell viability, spheroid formation, and reduces levels of cyclin A and CD44. Taken together, these findings suggest that autophagy may promote tumor progression and confer therapeutic resistance in this malignancy 99.

In summary, the current evidence reveals autophagy as a dynamic process in endometrial cancer that can either inhibit or promote tumor progression depending on specific molecular contexts. These insights highlight the need for precisely targeted autophagy modulation strategies in therapeutic development.

## Discussion and Perspectives

As a fundamental cellular process governing cell survival, metabolism, hormonal and immuno balance, autophagy plays a critical yet complex role in endometrial pathophysiology. While maintaining cellular homeostasis in healthy endometrium, its dysregulation contributes significantly to disease progression, exhibiting context-dependent effects across various endometrial disorders.

Despite significant progress in elucidating the role of autophagy in endometrial diseases, several key unresolved questions remain. One primary challenge is the lack of standardized and accurate methodologies for monitoring autophagic activity. A significant number of studies rely heavily on static techniques such as immunoblotting or histological analysis of LC3, or on pharmacological inhibitors like CQ and 3-MA. These approaches offer limited insight into the dynamic process of autophagic flux, unlike more advanced tools such as tandem fluorescent LC3 reporters. The absence of reliable and uniformly applied detection methods may lead to inconsistent and potentially paradoxical data, hindering the extraction of functional conclusions and insights.

Current understanding of autophagy's role in endometrial diseases remains incomplete, particularly regarding its precise crosstalk with hormonal signaling, inflammation and other cellular pathways. Besides, the functional role of autophagy in endometrial pathophysiology is profoundly context-dependent. Autophagic flux is highly dynamic, fluctuating throughout the menstrual cycle and across different stages of disease, yet its precise roles in these physiological and pathological processes remain poorly defined 100.

Furthermore, therapeutic development of autophagy-modulating agents also faces significant challenges, including the lack of specificity and potential side effects. For instance, while mTOR inhibitors like rapamycin show promise in preclinical studies, their clinical translation is hindered by potential systemic side effects. Additionally, the dynamic nature of autophagy, with its activation or inhibition could yield opposing outcomes, further complicate therapeutic strategies, demanding context-dependent approaches. These limitations underscore the need for more precise pharmacological interventions that can selectively target autophagic processes in disease-specific context of endometrial tissues.

Future research should focus on unraveling the tissue-specific regulation of autophagy in endometrial diseases, using advanced technologies such as single-cell sequencing and organoid models to dissect its spatiotemporal dynamics. Moreover, the exploration of combination therapies integrating autophagy modulators with existing treatments like hormones may enhance efficacy while minimizing resistance. Addressing these gaps will not only enhance our mechanistic understanding of autophagy in endometrial disorders but also facilitate the translation of these findings into clinically relevant therapeutic strategies, ultimately improving patient outcomes in this challenging field of women's health.

## Figures and Tables

**Figure 2:**
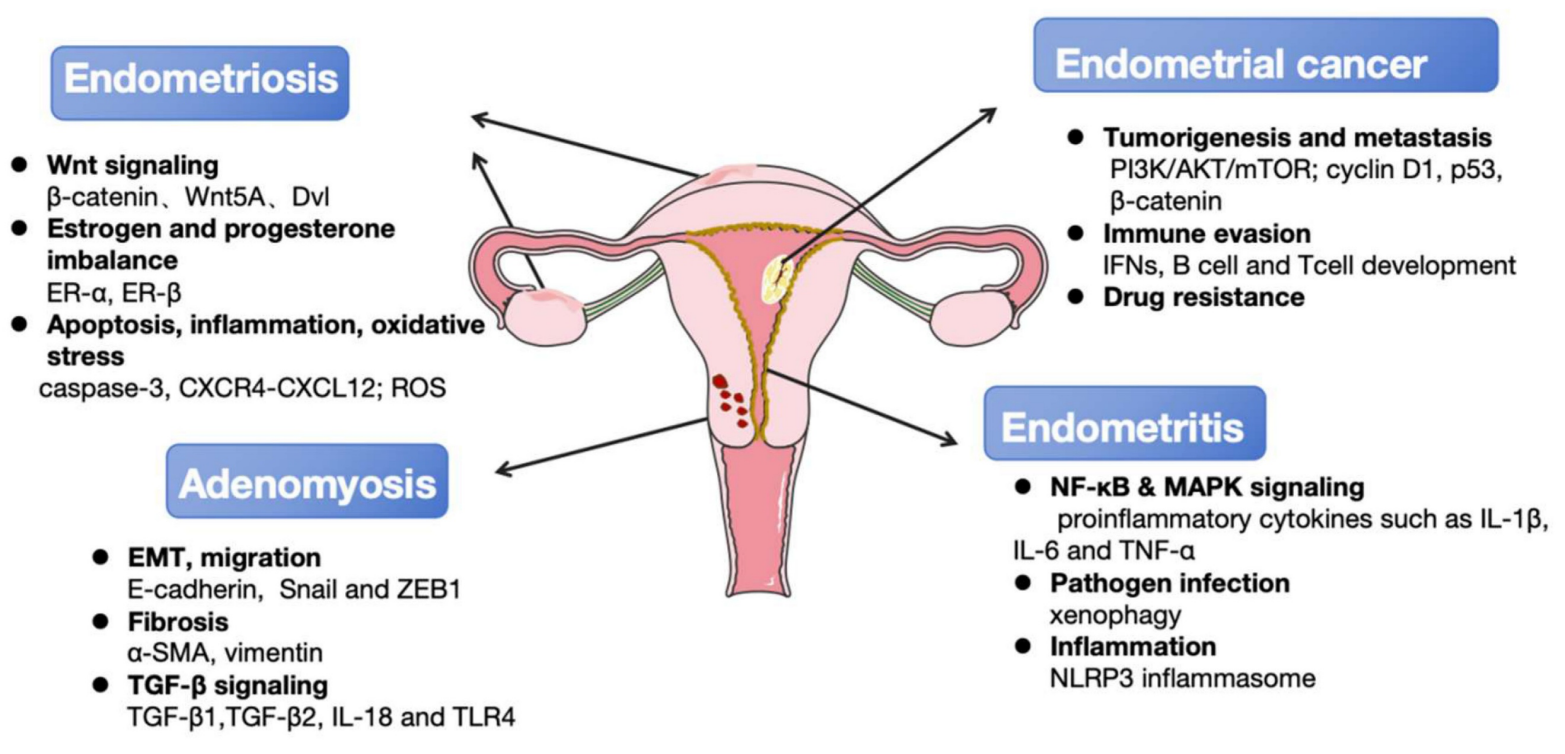
The principal autophagy-associated regulatory mechanisms involved in the occurrence and development of endometrial diseases. Multiple key signaling pathways (Wnt, NF-κB, MAPK, *ect.*) and cellular processes (apoptosis, EMT, fibrosis, *ect.*) are dysregulated in endometrial diseases. These interconnected mechanisms drive core disease features including local tumorigenesis, pain, and infertility. Furthermore, they increase the risk of malignant transformation to endometrial cancer and the development of comorbid gynecological disorders such as adenomyosis and endometritis. Key molecular signatures associated with disease progression were also listed.
